# Kingella kingae Surface Polysaccharides Promote Resistance to Neutrophil Phagocytosis and Killing

**DOI:** 10.1128/mBio.00631-19

**Published:** 2019-06-25

**Authors:** Vanessa L. Muñoz, Eric A. Porsch, Joseph W. St. Geme

**Affiliations:** a Department of Microbiology, University of Pennsylvania Perelman School of Medicine, Philadelphia, Pennsylvania, USA; b Department of Pediatrics, The Children's Hospital of Philadelphia, Philadelphia, Pennsylvania, USA; New York University; University at Buffalo SUNY; University of Virginia

**Keywords:** *Kingella kingae*, capsule, exopolysaccharide, immune evasion, neutrophils

## Abstract

Bacterial pathogens have evolved strategies that enable them to evade neutrophil-mediated killing. The Gram-negative coccobacillus Kingella kingae is an emerging pediatric pathogen and is increasingly recognized as a common etiological agent of osteoarticular infections and bacteremia in young children. K. kingae produces a polysaccharide capsule and an exopolysaccharide, both of which are important for protection against complement-mediated lysis and are required for full virulence in an infant rat model of infection. In this study, we examined the role of the K. kingae polysaccharide capsule and exopolysaccharide in protection against neutrophil killing. In experiments with primary human neutrophils, we found that the capsule interfered with the neutrophil oxidative burst response and prevented neutrophil binding of K. kingae but had no effect on neutrophil internalization of K. kingae. In contrast, the exopolysaccharide resisted the bactericidal effects of antimicrobial peptides and efficiently blocked neutrophil phagocytosis of K. kingae. This work demonstrates that the K. kingae polysaccharide capsule and exopolysaccharide promote evasion of neutrophil-mediated killing through distinct yet complementary mechanisms, providing additional support for the K. kingae surface polysaccharides as potential vaccine antigens. In addition, these studies highlight a novel interplay between a bacterial capsule and a bacterial exopolysaccharide and reveal new properties for a bacterial exopolysaccharide, with potential applicability to other bacterial pathogens.

## Abstract (precis)

Kingella kingae is a Gram-negative commensal in the oropharynx and represents a leading cause of joint and bone infections in young children. The mechanisms by which K. kingae evades host innate immunity during pathogenesis of disease remain poorly understood. In this study, we established that the K. kingae polysaccharide capsule and exopolysaccharide function independently to protect K. kingae against reactive oxygen species (ROS) production, neutrophil phagocytosis, and antimicrobial peptides. These results demonstrate the intricacies of K. kingae innate immune evasion and provide valuable information that may facilitate development of a polysaccharide-based vaccine against K. kingae.

## INTRODUCTION

Kingella kingae, a Gram-negative coccobacillus, is a common commensal organism in the oropharynx of young children (1). Despite being an oral commensal, K. kingae is a primary cause of osteoarticular infections and a common etiology of bacteremia in children between 6 and 36 months of age (2–4). Recent studies have elucidated K. kingae surface and secreted factors that promote virulence via adherence to epithelial cells, cytotoxicity, and immune evasion (5–10).

To colonize the oropharynx and survive in the hostile intravascular environment, K. kingae must evade innate immunity. K. kingae produces a polysaccharide capsule and a galactan homopolymer exopolysaccharide, both of which have been demonstrated to contribute to virulence in an infant rat infection model (10–12). Recent work has determined that the K. kingae polysaccharide capsule and exopolysaccharide confer high-level resistance to human serum (10). Elimination of both surface polysaccharides is detrimental to the organism in the presence of human serum, resulting in increased deposition of antibodies and complement fragments and, ultimately, complement activation and bacterial lysis (10).

Neutrophils are the most abundant leukocyte type in the blood and the predominant infiltrating leukocyte type during acute inflammation (13). These cells mobilize to clear pathogenic bacteria through various extracellular and intracellular mechanisms and are primed and activated by a variety of inflammatory stimulants, including conserved bacterial ligands known as pathogen-associated molecular patterns (PAMPs). PAMPS are recognized by membrane-associated Toll-like receptors (TLRs), and subsequent TLR activation primes neutrophils and promotes phagocytosis, degranulation, and production of reactive oxygen species (ROS).

Given the role of neutrophils in combating microbial invaders, bacteria have evolved multiple mechanisms to evade neutrophil-mediating killing. Encapsulation by invasive pathogens such as Streptococcus pneumoniae and Neisseria meningitidis has been demonstrated to promote bacterial survival by inhibiting neutrophil recognition and activation (14–16). Encapsulation has also been demonstrated to prevent antibody recognition of surface antigens present on the bacterial surface and to inhibit complement deposition and activation (16–19). Opsonization by immunoglobulins and complement components augments neutrophil recognition and enhances neutrophil antimicrobial activity, including phagocytosis of opsonized bacteria.

In this study, we found that the K. kingae polysaccharide capsule promotes neutrophil evasion by preventing neutrophil activation, dampening ROS production, and inhibiting initial neutrophil binding of K. kingae. Interestingly, we observed a distinctive role for the exopolysaccharide in promoting K. kingae survival in the presence of neutrophil antimicrobial peptides and in blocking neutrophil phagocytosis of bound bacteria. The absence of both the polysaccharide capsule and the exopolysaccharide increased neutrophil opsonophagocytosis of K. kingae. This study demonstrates the importance of the K. kingae polysaccharide capsule and exopolysaccharide in neutrophil evasion, presumably promoting hematogenous dissemination of K. kingae.

## RESULTS

### Capsule and exopolysaccharide prevent neutrophil-mediated killing of K. kingae.

**FIG 1:**
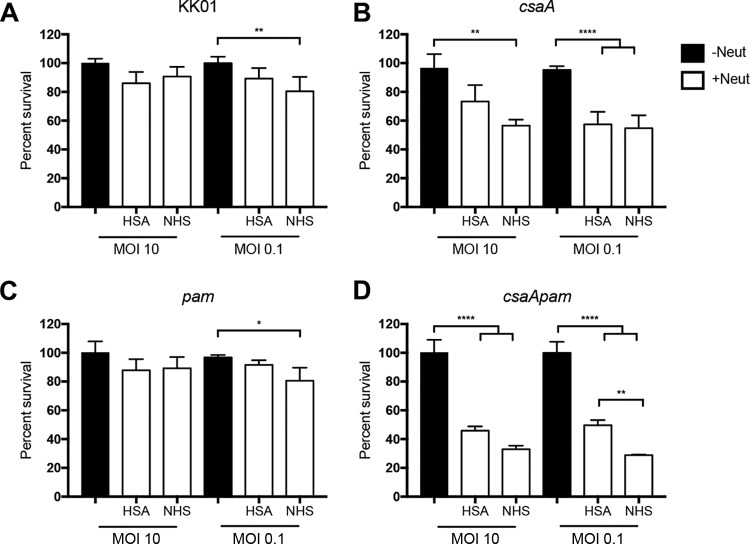
Elimination of the K. kingae capsule results in K. kingae killing by neutrophils. (A to D) K. kingae strains KK01, KK01 *csaA*, KK01 *pam*, and KK01 *csaA pam*, as indicated, were incubated with (+) or without (−) human neutrophils at an MOI of 10 or 0.1 for 1 h in the presence of 1% HSA or 1% NHS. Survival was determined by dividing the number of recovered CFU by the number of CFU in the inoculum. A total of three biological replicates were performed (*n* = 3). Statistical significance was determined using two-way analysis of variance and a Tukey *post hoc* test. *, *P < *0.05; **, *P < *0.01; ****, *P < *0.0001.

In previous work we demonstrated that the polysaccharide capsule and exopolysaccharide expressed by K. kingae protect the organism from complement-mediated lysis and promote virulence in an infant rat model of infection (10–12). To further characterize the role of these surface polysaccharides in innate immune evasion, we performed neutrophil-killing assays using K. kingae strain KK01, the capsule-deficient mutant KK01 *csaA* (12), the exopolysaccharide-deficient mutant KK01 *pam* (11), and the capsule-deficient and exopolysaccharide-deficient mutant KK01 *csaA pam* (10). Using a multiplicity of infection (MOI) of 0.1 or 10, the K. kingae strains were incubated with or without purified human neutrophils for 1 h in the presence of 1% normal human serum (NHS) as a source of serum opsonins or 1% human serum albumin (HSA) as a control. To assess extracellular and intracellular survival of K. kingae in the presence of neutrophils, supernatant and neutrophil lysates were diluted and plated to determine CFU counts. Survival percentage was calculated by dividing the number of recovered CFU by the number of inoculated CFU. Elimination of the capsule, the exopolysaccharide, or both the capsule and the exopolysaccharide resulted in no growth defects on solid agar (10) and had no effect on survival in the absence of neutrophils or 1% NHS alone (Fig. 1, black bars; see also Fig. S1 in the supplemental material). Survival rates of strains KK01 and KK01 *pam* were only slightly affected at an MOI of 0.1 in the presence of neutrophils with 1% NHS (Fig. 1A and C). Survival of strain KK01 *csaA* was reduced in the presence of neutrophils with 1% NHS at both MOIs and in the presence of neutrophils with 1% HSA at an MOI of 0.1, with ∼60% survival (Fig. 1B). Interestingly, survival of strain KK01 *csaA pam* was markedly reduced in the presence of neutrophils at both MOIs (Fig. 1D). In contrast to the other strains tested, strain KK01 *csaA pam* was more susceptible to neutrophil-mediated killing when opsonins were present, with ∼30% survival with opsonins and ∼50% survival without opsonins (Fig. 1D). These results establish that the capsule is critical for K. kingae neutrophil evasion and that the exopolysaccharide plays a conditional protective role when opsonins are present and the capsule is absent.

10.1128/mBio.00631-19.1 FIG S1 Survival of K. kingae in the presence of 1% normal human serum. K. kingae strains KK01, KK01 *csaA*, KK01 *pam*, and KK01 *csaA pam* were incubated with 1% normal human serum at an MOI equivalent of 0.1 (white bars) or 10 (black bars) for 1 h in the absence of neutrophils. Survival was determined by dividing the recovered CFU by the inoculum CFU. A total of three biological replicates were performed (*n* = 3). Download FIG S1, TIF file, 0.04 MB. Copyright © 2019 Muñoz et al. 2019 Muñoz et al. This content is distributed under the terms of the Creative Commons Attribution 4.0 International license.

### The K. kingae capsule inhibits the neutrophil oxidative burst response.

**FIG 2:**
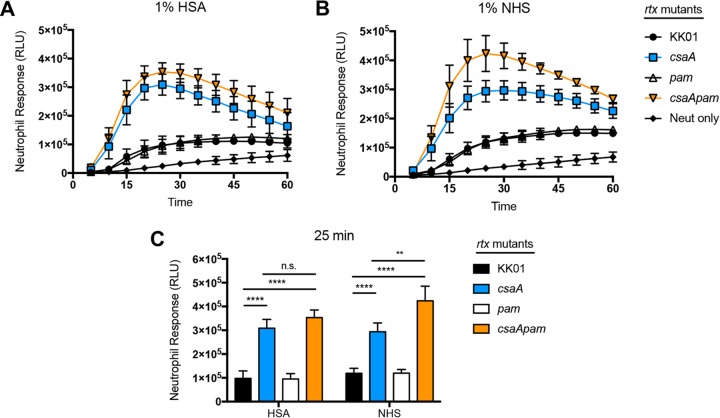
The absence of the K. kingae capsule results in an enhanced neutrophil oxidative burst response. K. kingae *rtx* mutant strains were incubated with human neutrophils at an MOI of 10 in the presence of 1% HSA or 1% NHS. Chemiluminescence was measured at 5-min intervals for 60 min for kinetic analysis (A and B) or at the 25-min time point (C). A total of three biological replicates were performed (*n* = 3). Statistical significance was determined using two-way analysis of variance and a Tukey *post hoc* test. RLU, relative light units; n.s., not significant; **, *P < *0.01; ****, *P < *0.0001.

The oxidative burst response generated by human neutrophils is a critical innate immune defense mechanism against bacterial pathogens. K. kingae expresses a cytotoxin called RtxA that is capable of rapidly killing a variety of human cell types, including leukocytes (5, 7, 9). *In vitro*, strains KK01 and KK01 *rtx* (a mutant with the *rtx* locus deleted) demonstrate similar survival rates in the presence of neutrophils at an MOI of 10 (Fig. S2). However, to eliminate the influence of the RtxA toxin as a confounding variable on neutrophil activation and ROS production in the presence of K. kingae, we deleted the *rtx* locus in strains KK01, KK01 *csaA*, KK01 *pam*, and KK01 *csaA pam*. To determine the oxidative burst response, we incubated the K. kingae *rtx* mutant strains at an MOI of 10 with human neutrophils and either 1% HSA or 1% NHS in the presence of luminol (Fig. 2A to C). Based on measurement of chemiluminescence (as relative light units [RLU]) at 5-min intervals over a 1-h incubation period, incubation of strains KK01 *rtx* and KK01 *pam rtx* with neutrophils resulted in a moderate neutrophil response compared to that of the uninfected control (Fig. 2A and B). In contrast, incubation of strains KK01 *csaA rtx* and KK01 *csaA pam rtx* resulted in a rapid and robust neutrophil response, peaking at the 25-min time point (Fig. 2A and B).

10.1128/mBio.00631-19.2 FIG S2 Elimination of RtxA toxin has no effect on K. kingae survival in the presence of neutrophils. K. kingae strains KK01 (A), KK01 *rtx* (A), KK01 *csaA* (B), KK01 *csaA rtx* (B), KK01 *pam* (C), KK01 *pam rtx* (C), KK01 *csaA pam* (D), and KK01 *csaA pam rtx* (D) were incubated with human neutrophils at an MOI of 10 for 1 h in the presence of 1% HSA (white bars) or 1% NHS (black bars). Survival was determined by dividing the recovered CFU by the inoculum CFU. A total of three biological replicates were performed (*n* = 3). Download FIG S2, TIF file, 0.1 MB. Copyright © 2019 Muñoz et al. 2019 Muñoz et al. This content is distributed under the terms of the Creative Commons Attribution 4.0 International license.

Using the 25-min time point, the neutrophil responses were compared across the KK01 *rtx* mutant strains in either 1% HSA or 1% NHS. In both 1% HSA and 1% NHS, we observed a statistically significant increase in the neutrophil response to strains KK01 *csaA rtx* and KK01 *csaA pam rtx* compared to the response to strain KK01 *rtx* (Fig. 2C). Comparison of strains KK01 *rtx* and KK01 *pam rtx* in 1% HSA or 1% NHS revealed no statistically significant difference in neutrophil responses (Fig. 2C). Similarly, comparison of strains KK01 *csaA rtx* and KK01 *csaA pam rtx* in 1% HSA revealed no statistically significant difference in neutrophil responses (Fig. 2C). In contrast, there was a statistically significant increase in the neutrophil response to strain KK01 *csaA pam rtx* compared to that to strain KK01 *csaA rtx* in 1% NHS (Fig. 2C). These data suggest that K. kingae encapsulation prevents neutrophil activation and ROS production and release. Moreover, the neutrophil response is most robust when both surface polysaccharides are absent and opsonins are present.

### Neutrophil-mediated killing of capsule-deficient K. kingae is dependent on phagocytosis and ROS production.

**FIG 3:**
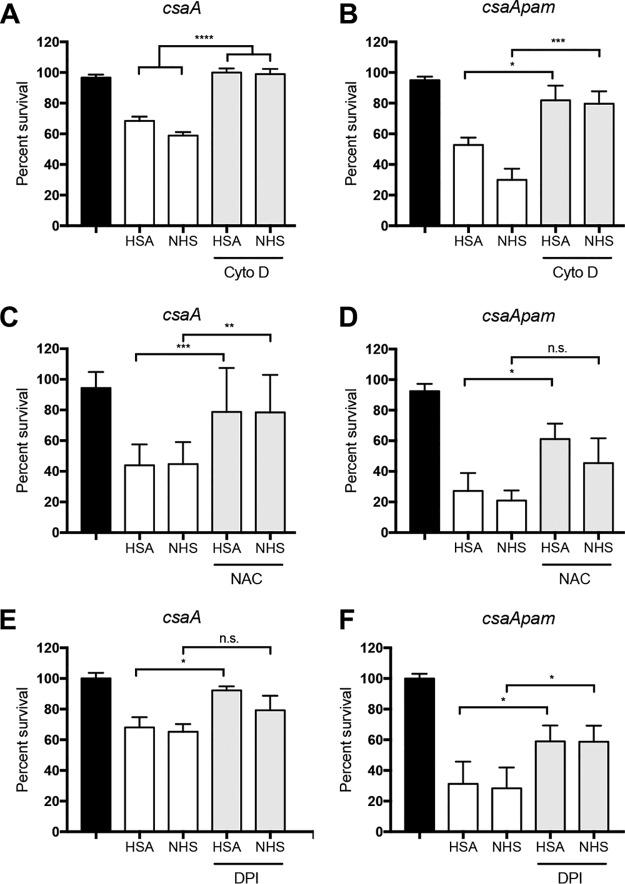
Inhibition of neutrophil phagocytosis and ROS production enhances survival of nonencapsulated K. kingae. K. kingae strains KK01 *csaA* and KK01 *csaA pam* were incubated with human neutrophils at an MOI of 0.1 for 30 min. Neutrophils were preincubated with 10 μg/ml cytochalasin D (CytoD) (A and B), 10 mM *N*-acetylcysteine (NAC) (C and D), or 4 μM diphenyleneiodonium (DPI) (E and F) in the presence of 1% HSA or 1% NHS. Black bars indicate survival in the absence of neutrophils, white bars represent survival in the presence of neutrophils, and gray bars represent survival in the presence of neutrophils pretreated as indicated. Survival was determined by calculating the number of recovered CFU by the number of CFU in the inoculum. A total of three biological replicates were performed (*n* = 3). Statistical significance was determined using two-way analysis of variance and a Tukey *post hoc* test. n.s., not significant; *, *P < *0.05; **, *P < *0.01; ***, *P < *0.001; ****, *P < *0.0001.

Neutrophils are recruited early during infection and can eliminate pathogens through a variety of mechanisms, including phagocytosis and the oxidative burst (20). To determine whether phagocytosis and/or ROS production facilitates killing of nonencapsulated K. kingae strains, we performed neutrophil-killing assays in the presence of cytochalasin D (CytoD), the antioxidant *N*-acetylcysteine (NAC), or the NADPH oxidase inhibitor diphenyleneiodonium (DPI). Using an MOI of 0.1, strains KK01 *csaA* and KK01 *csaA pam* were incubated with human neutrophils pretreated with CytoD, NAC, or DPI for 30 min. Pretreatment of neutrophils with CytoD restored survival of strains KK01 *csaA* and KK01 *csaA pam* in the presence of neutrophils (Fig. 3A and B). Pretreatment of neutrophils with NAC led to a roughly 2-fold increase in the percent survival for strains KK01 *csaA* and KK01 *csaA pam* in 1% HSA and 1% NHS but did not completely restore survival to the level observed with neutrophils pretreated with vehicle only (Fig. 3C and D). Similarly, pretreatment of neutrophils with DPI led to a significant increase in the percent survival for strain KK01 *csaA* in 1% HSA (Fig. 3E). Pretreatment of neutrophils with DPI led to a roughly 2-fold increase in the percent survival for strain KK01 *csaA pam* but did not completely restore survival (Fig. 3F). These data suggest that phagocytosis and ROS are key mechanisms contributing to neutrophil-mediated killing of nonencapsulated K. kingae.

### The K. kingae exopolysaccharide protects against neutrophil antimicrobial peptides.

**FIG 4:**
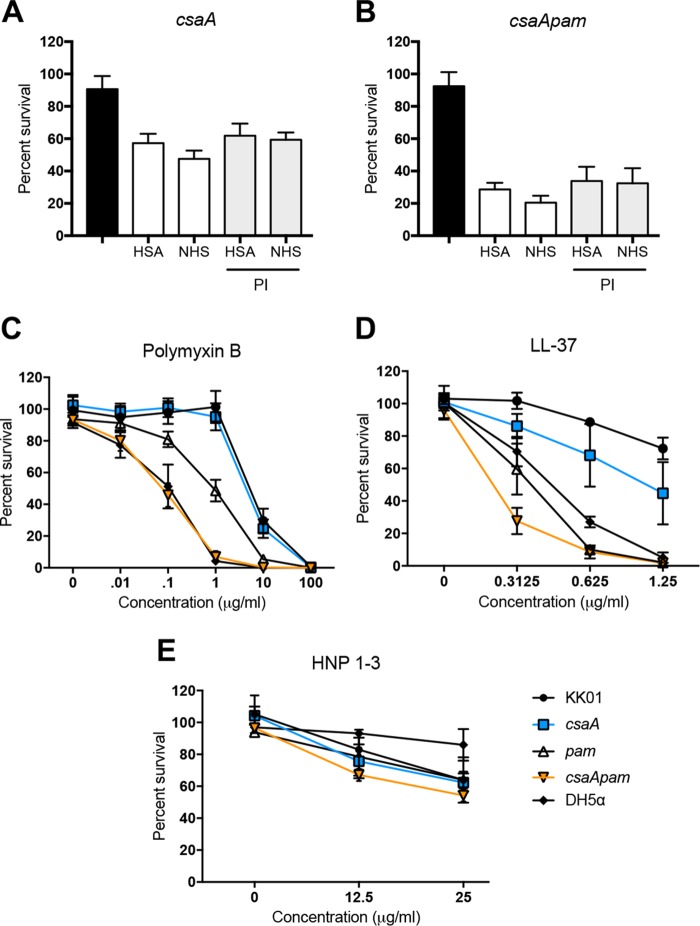
The surface polysaccharides protect K. kingae against antimicrobial peptides. K. kingae strains KK01 *csaA* and KK01 *csaA pam* were incubated with human neutrophils preincubated with a protease inhibitor (PI) cocktail at an MOI of 0.1 for 30 min in the presence of 1% HSA or 1% NHS (A and B). Black bars indicate survival in the absence of neutrophils, white bars represent survival in the presence of neutrophils, and gray bars represent survival in the presence of neutrophils pretreated with PI. (C to E) K. kingae strains and E. coli strain DH5α were incubated with various concentrations of antimicrobial peptides (polymyxin B, cathelicidin LL-37, or HNP-1 to HNP-3), as indicated, for 30 min. A total of three biological replicates were performed (*n* = 3). Survival was determined by calculating the number of recovered CFU by the number of CFU in the inoculum.

Neutrophil granules are cytoplasmic vesicles that can fuse with phagosomes or the plasma membrane to release degradative enzymes (e.g., myeloperoxidase, elastase, and cathepsin G), antimicrobial peptides (e.g., defensins and the cathelicidin LL-37), and reactive radicals to kill intracellular and extracellular microorganisms (20). To determine whether proteases released during degranulation facilitate killing of K. kingae, we performed neutrophil-killing assays in the presence of a protease inhibitor (PI) cocktail. Using an MOI of 0.1, strains KK01 *csaA* and KK01 *csaA pam* were incubated with PI-pretreated human neutrophils for 30 min. Pretreatment of neutrophils with PI had no statistically significant effect on levels of survival for strains KK01 *csaA* and KK01 *csaA pam* (Fig. 4A and B). To determine the sensitivity of K. kingae in the presence of antimicrobial peptides, we performed bactericidal assays with a bacterial peptide, polymyxin B, and human peptides, LL-37 and HNP-1 to HNP-3, at a range of physiologic concentrations. Survival of strain KK01 *csaA* compared to that of strain KK01 was not affected in the presence of polymyxin B and was slightly decreased in the presence of LL-37 (Fig. 4C and D). Interestingly, survival of strain KK01 *pam* compared to that of strain KK01 was significantly decreased in the presence of polymyxin B or LL-37 (Fig. 4C and D). Strain KK01 *csaA pam* was more sensitive in the presence of polymyxin B and LL-37 than strain KK01 (Fig. 4C and D). We observed no change in survival of the mutant strains compared to that of strain KK01 in the presence of HNP-1 to HNP-3 (Fig. 4E). These results establish that the exopolysaccharide is critical for survival of K. kingae in the presence of antimicrobial peptides and demonstrate a moderate role for the capsule.

### The K. kingae surface polysaccharides inhibit bacterial association and phagocytosis by neutrophils.

**FIG 5:**
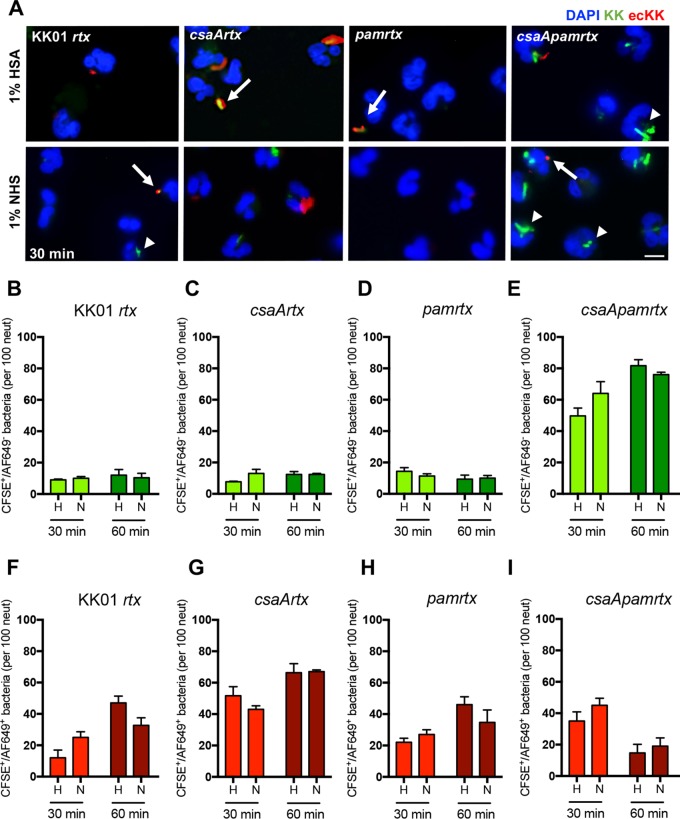
The presence of the K. kingae surface polysaccharides results in reduced neutrophil association and phagocytosis of K. kingae. Immunofluorescence images (A) are shown along with quantitative analyses (B to I) of K. kingae *rtx* mutant strains incubated with human neutrophils at an MOI of 10 for 30 min or 60 min, as indicated, in the presence of 1% HSA or 1% NHS. K. kingae strains were stained with CFSE (green) prior to infection. Neutrophils were fixed and incubated with a guinea pig anti-K. kingae antiserum and subsequently with anti-guinea pig Alexa Fluor-649 (AF649) to detect extracellular bacteria (red); 4′,6′-diamidino-2-phenylindole (DAPI) was used to stain the nuclei of the neutrophils (blue). Images were acquired using a 20× objective. The arrows indicate extracellular bacteria, and the arrowheads indicate intracellular bacteria. Scale bar, 5 μm. The graphs depict the total number of intracellular (B to E) bacteria and the total number of extracellular (F to I) bacteria for the indicated strains. The data represent the total number of CFSE^+^ events per 100 neutrophils chosen randomly per biological replicate, and a total of three biological replicates were performed (*n* = 3). Abbreviations: H, HSA; N, NHS; KK, K. kingae; ecKK, extracellular K. kingae.

To further delineate the mechanism of neutrophil-mediated killing of K. kingae strains, we performed immunofluorescence microscopy, examining the number of K. kingae bacteria associated with neutrophils and the nature of the association. K. kingae *rtx* mutant strains were used to prevent cell lifting during infection. K. kingae strains were stained with carboxyfluorescein succinimidyl ester (CFSE) prior to infection and were incubated at an MOI of 10 with human neutrophils for 30 min or 60 min in the presence of 1% HSA or 1% NHS. The wells were washed vigorously to remove any unassociated bacteria, and the samples were incubated with an anti-K. kingae whole-organism guinea pig antiserum to detect extracellular bacteria associated with the neutrophils. For the 30-min time point, the total number of associated bacteria (intracellular and extracellular) was 2.8-fold higher for strain KK01 *csaA rtx* than for strain KK01 *rtx* in 1% HSA and 1.6-fold higher for strain KK01 *csaA rtx* than for to strain KK01 *rtx* in 1% NHS (Fig. 5A and Table 1). The total number of associated bacteria was 4.0-fold higher for strain KK01 *csaA pam rtx* than for strain KK01 *rtx* in 1% HSA and 3.1-fold higher for strain KK01 *csaA pam rtx* than for strain KK01 *rtx* in 1% NHS (Fig. 5A; Table 1). While we observed an increase in total bacterial association with strain KK01 *csaA rtx* compared to that with strain KK01 *rtx*, there was no significant difference between the numbers of intracellular bacteria (Fig. 5B and C; Table 1). In contrast, we observed a significant increase in the number of intracellular KK01 *csaA pam rtx* compared to levels for strains KK01 *rtx*, KK01 *csaA rtx*, and KK01 *pam rtx* in 1% HSA and 1% NHS (Fig. 5B to E). We also observed a statistically significant increase in the total number of associated bacteria for strain KK01 *csaA pam rtx* in 1% NHS compared to that in 1% HSA (Table 1).

**TABLE 1:** Total number of CFSE^+^ bacteria associated with human neutrophils

Strain	No. of bacteria (mean ± SD) by treatment group:^*a*^			
	1% HSA		1% NHS	
	30 min	60 min	30 min	60 min
KK01 *rtx*	21 ± 8.2	59 ± 9.2	35 ± 7.0	43 ± 13.1
KK01 *csaA rtx*	59 ± 9.5	79 ± 7.1	56 ± 3.6	79 ± 2.3
KK01 *pam rtx*	36 ± 5.5	58 ± 5.9	38 ± 3.2	45 ± 11.7
KK01 *csaA pam rtx*	85 ± 9.0	96 ± 9.0	109 ± 7.8	95 ± 9.5

a The data represent the total number of CFSE^+^ events per 100 neutrophils chosen randomly per replicate. A total of three biological replicates were performed.

At the 60-min time point, we observed an increase in total number of associated bacteria for strains KK01 *rtx*, KK01 *csaA rtx*, and KK01 *pam rtx*; however, the numbers of intracellular bacteria remained similar across the 30-min and 60-min time points (Table 1; Fig. 5B, D, and F to H). We observed a slight increase in the total number of associated bacteria for strain KK01 *csaA pam rtx* at 60 min (Table 1). Comparing levels at the 60-min time point to those at the 30-min time point, we observed a significant decrease in the number of extracellular bacteria and a slight increase in the number of intracellular bacteria for strain KK01 *csaA pam rtx* (Fig. 5E and I).

Taken together, these results demonstrate that the absence of capsule results in increased bacterial association with neutrophils and that only the elimination of both the capsule and the exopolysaccharide results in a significant increase of K. kingae phagocytosis by neutrophils, regardless of opsonin deposition.

## DISCUSSION

**FIG 6:**
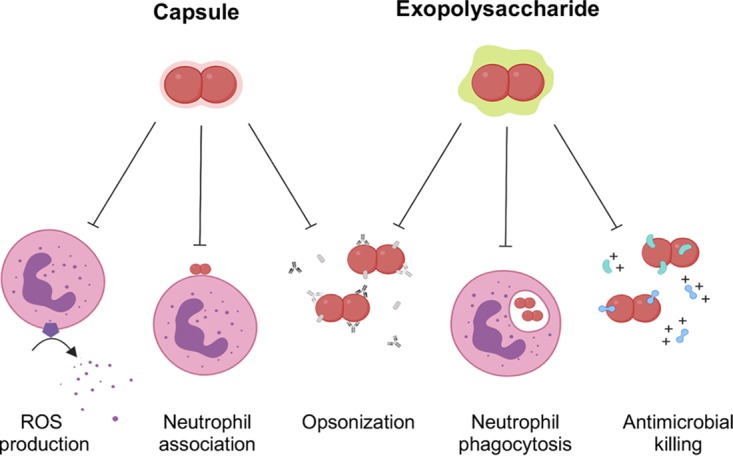
K. kingae polysaccharide capsule and exopolysaccharide are multifunctional surface structures that facilitate neutrophil evasion. K. kingae polysaccharide capsule and exopolysaccharide function distinctly to promote neutrophil evasion. The K. kingae polysaccharide capsule prevents ROS production and neutrophil association while the K. kingae exopolysaccharide reduces neutrophil phagocytosis and sensitivity to antimicrobial peptides. The presence of either the capsule or the exopolysaccharide blocks complement and antibody opsonization.

The pathogenesis of K. kingae disease involves bacterial invasion of the bloodstream, evasion of complement- and neutrophil-mediated killing, and dissemination to distant sites (1, 5, 21–23). The K. kingae surface polysaccharides have proven to be critical for complement evasion, and expression of both the capsule and the exopolysaccharide is essential for full virulence in the infant rat model of infection (10). In this study, we observed that in the absence of the polysaccharide capsule, K. kingae was sensitive to neutrophil-mediated killing. This phenotype was enhanced when the exopolysaccharide was also absent. Further analysis demonstrated that the polysaccharide capsule prevented neutrophil ROS production and interfered with neutrophil binding of K. kingae but had a limited effect on resistance to neutrophil antimicrobial peptides and no direct effect on phagocytosis. In contrast, the exopolysaccharide conferred resistance to neutrophil antimicrobial peptides and blocked neutrophil phagocytosis but had a limited effect on neutrophil binding of K. kingae. Overall, we have established a novel interplay between the K. kingae polysaccharide capsule and exopolysaccharide, which have complementary but distinct roles in mediating resistance to human neutrophils (Fig. 6).

Polysaccharide capsules expressed by invasive pathogens such as N. meningitidis and S. pneumoniae promote bacterial survival during infection by interfering with neutrophil recognition, activation, and phagocytosis (16, 24, 25). In this study, we demonstrated that the presence of the K. kingae polysaccharide capsule significantly reduced the oxidative burst response by neutrophils. Many encapsulated pathogens prevent ROS production by inhibiting serum opsonization (16–18). In the case of K. kingae, elimination of the polysaccharide capsule increased ROS production regardless of the presence of human serum. Interestingly, only elimination of both the polysaccharide capsule and the exopolysaccharide led to a significant increase in ROS production when opsonins were present. These data are consistent with our previous study demonstrating that the presence of either the polysaccharide capsule or the exopolysaccharide was sufficient to prevent opsonization of K. kingae (10). Our results suggest that encapsulation prevents ROS production through mechanisms beyond hindering opsonin deposition. We hypothesize that when the K. kingae polysaccharide capsule is absent, increased neutrophil recognition of bacterial lipopolysaccharide (LPS) prompts TLR4 activation. TLR4 is activated by LPS and upon activation recruits the NADPH-oxidase complex, Nox4, leading to the generation of ROS (26–29). Future studies will assess the stimulatory potential of K. kingae LPS.

Aside from preventing ROS production, the K. kingae polysaccharide capsule decreased the total number of neutrophil-associated bacteria. It is well documented that bacterial polysaccharide capsules prevent neutrophil association by blocking opsonization (16–18). Interestingly, in our studies neutrophil binding of K. kingae occurred regardless of opsonization, which is similar to ROS production. Studies have shown nonopsonic neutrophil interactions by Neisseria gonorrhoeae and Helicobacter pylori through the expression of adhesins on the bacterial surface (30–33). In our previous work, we established that the K. kingae polysaccharide capsule can interfere with adherence to epithelial cells (8). In the absence of type IV pili, the polysaccharide capsule masks the trimeric autotransporter, Knh, and deletion of the capsule locus restores Knh-mediated adherence to wild-type levels (8). Neutrophil-killing assays have not been performed using Knh-deficient or pilus-deficient K. kingae strains to determine whether association with neutrophils is affected by these adhesins.

It is noteworthy that despite an increase in the total number of bound bacteria, the elimination of capsule did not increase phagocytosis regardless of opsonization. This observation suggests that the K. kingae exopolysaccharide inhibits or delays neutrophil phagocytosis. Exopolysaccharide secretion in other bacterial pathogens is commonly associated with biofilm formation, promoting survival in the host by inhibiting opsonization and phagocytosis (34–36). Based on previous studies, we presume that the K. kingae exopolysaccharide remains loosely tethered to the K. kingae cell surface, at least in part (10, 11). Surface analysis using scanning electron microscopy may be beneficial for identifying structural or physical changes in the K. kingae cell surface that alter interactions with human neutrophils to inhibit or reduce neutrophil phagocytosis. Alternatively, the presence of the exopolysaccharide on the K. kingae bacterial surface may mask surface antigens that are recognized by neutrophil receptors and trigger phagocytosis.

Cationic antimicrobial peptides function through electrostatic interactions with anionic bacterial surfaces (37–40). While bacterial polysaccharide capsules vary in their polymer composition, most capsules are anionic and have been shown to bind antimicrobial peptides to prevent or reduce undesirable interactions with the bacterial surface (14, 24, 25). Surprisingly, there are cases where the lack or loss of the polysaccharide capsule increases resistance to antimicrobial peptides, with examples including S. pneumoniae and Campylobacter jejuni (15, 41). Given that the K. kingae polysaccharide capsule blocks antibody recognition of surface antigens (10), we hypothesized that the capsule would be necessary for complete protection against antimicrobial peptides. Interestingly, loss of the polysaccharide capsule had no effect on K. kingae survival in the presence of polymyxin B and only slightly affected survival in the presence of cathelicidin LL-37. In contrast, elimination of the exopolysaccharide resulted in a marked decrease in K. kingae survival in the presence of increasing concentrations of either polymyxin B or LL-37 compared to levels in the wild-type strain, suggesting that the exopolysaccharide is essential for antimicrobial peptide resistance. Llobet et al. demonstrated that free capsular material shed or purified from K. pneumoniae, S. pneumoniae, or Pseudomonas aeruginosa acted as a decoy by sequestering and neutralizing polymyxin B and HNP-1 (42). The secretion and release of the high-molecular-weight exopolysaccharide by K. kingae may provide a physical barrier and also actively scavenge antimicrobial peptides to protect K. kingae more efficiently than the polysaccharide capsule. Similar to K. kingae survival in the presence of neutrophils, elimination of both the capsule and the exopolysaccharide led to the most sensitive phenotype in assays with antimicrobial peptides, suggesting that the capsule plays a minor role in protection when the exopolysaccharide is absent.

Given the robust oxidative burst response and phagocytic activities of neutrophils in assays with surface polysaccharide-deficient K. kingae, pretreatment of neutrophils with ROS inhibitors, DPI, and NAC or with an actin polymerase inhibitor, CytoD, restored survival of K. kingae mutants to wild-type levels. Together, our data suggest that killing of the capsule-deficient K. kingae strains is predominately due to ROS production, whereas the enhanced killing of the capsule-deficient and exopolysaccharide-deficient K. kingae is meditated through both ROS production and phagocytosis. Furthermore, degranulation and release of antimicrobial peptides by neutrophils may augment killing of exopolysaccharide-deficient K. kingae.

In our earlier work, we observed redundancy of the K. kingae polysaccharide capsule and exopolysaccharide in preventing antibody and complement deposition on the bacterial surface (10). In this study, we observed largely distinct but interdependent roles for the polysaccharide capsule and the exopolysaccharide in protecting K. kingae from neutrophil-mediated killing and antimicrobial peptides. This study demonstrates the protective functions of the K. kingae polysaccharide capsule and exopolysaccharide against human neutrophils and highlights the robust and multifaceted mechanisms employed by K. kingae to evade innate immunity.

## MATERIALS AND METHODS

### Bacterial strains and growth conditions.

The strains used in this study are listed in Table 2. K. kingae strains were stored at −80°C in brain heart infusion (BHI) broth with 20% glycerol. The Escherichia coli strains were stored at −80°C in Luria-Bertani (LB) broth with 15% glycerol. K. kingae strains were grown at 37°C with 5% CO_2_ on chocolate agar plates supplemented with 50 μg/ml kanamycin or 1 μg/ml erythromycin, as appropriate. E. coli strains were grown at 37°C on LB agar or shaking at 250 rpm in LB broth supplemented with 100 μg/ml ampicillin or 50 μg/ml kanamycin, as appropriate.

**TABLE 2:** Strains and plasmid used in this study

Strain or plasmid	Description	Reference or source
*K. kingae* strains		
KK01	Nonspreading/noncorroding derivative of 269-492	5
KK01 *csaA*	KK01 with *csaA* deletion	12
KK01 *pam*	KK01 with *pamABCDE* deletion	11
KK01 *csaA pam*	KK01 with *csaA* deletion and *pamABCDE* deletion	10
KK01 *rtx*	KK01 with *rtxBDCA* deletion	This study
KK01 *csaA rtx*	KK01 with *csaA* deletion and *rtxBDCA* deletion	This study
KK01 *pam rtx*	KK01 with *pam* deletion and *rtxBDCA* deletion	This study
KK01 *csaA pam rtx*	KK01 with *csaA* deletion, *pam* deletion, and *rtxBDCA* deletion	This study
*E. coli* strain		
DH5α	λ^−^ ϕ80d*lacZ*ΔM15 Δ(*lacZYA*-*argF*)*U169 recA1 endA1 hsdR17*(r_K_^−^ m_K_^−^) *supE44 thi-1 gyrA relA1*	47
Plasmid		
pUC19*rtxBDCA*::*kan*	pUC19 with *aphA3* kanamycin cassette flanked by surrounding 5′ and 3′ regions of the *rtxBDCA* locus	This study

### Strain construction.

Strains KK01 *csaA*, KK01 *pam*, and KK01 *csaA pam* were generated as previously described (10–12). Briefly, plasmid-based gene disruption constructs were created in E. coli, linearized, and introduced into K. kingae using natural transformation. Transformants were recovered by selectively plating on chocolate agar plates with the appropriate antibiotic. Gene disruptions were confirmed by PCR.

As a reflection of the challenges in manipulating the K. kingae genome, we were unable to generate complementation constructs for strains KK01 *csaA* and KK01 *pam.* However, quantitative reverse transcription-PCR (qRT-PCR) analysis of the *hemB* gene downstream of *csaA* and the hypothetical gene (*hyp*) downstream of the *pam* locus demonstrated no evidence of polar effects, using the *ftsZ* housekeeping gene as the reference gene (mean fold change for *hemB*, 1.68 [*P > *0.05]; mean fold change for *hyp*, 0.92 [*P > *0.05]). While this analysis indicates no polar effects of the *csaA* and *pam* mutations, we cannot exclude the possibility of nonspecific effects of these mutations.

To generate pUC19*rtxBDCA*::*kan*, DNA fragments of the homologous recombination targeting sequence corresponding to the ∼1 kb upstream of *rtxB* and the ∼1 kb downstream of *rtxA* were PCR amplified from strain KK01 genomic DNA using primers rtx 5′ For/rtx 5′ Rev and rtx 3′ For/rtx 3′ Rev, respectively, and were cloned into pUC19 (Table 3). A kanamycin resistance cassette was then ligated into the BamHI site located between the cloned upstream and downstream homologous recombination targeting sequences. The plasmid was linearized with NdeI and was transformed into strain KK01, KK01 *csaA*, KK01 *pam*, and KK01 *csaA pam* via natural transformation. Transformants were recovered on chocolate agar plates containing 50 μg/ml kanamycin. Correct insertion of the gene disruption construct was confirmed via PCR and sequencing of the deletion site.

**TABLE 3:** Primers used in this study^*a*^

Primer	Sequence (5′–3′)
rtx 5′ For	GCAGGAATTCAATCTTGCGAATTTGGTGTG
rtx 5′ Rev	GCACGGATCCTCGGGCTGATAAATGCCGAC
rtx 3′ For	GCACGGATCCAGCTGAACAACGATTCAATG
rtx 3′ Rev	GCAGAAGCTTAGAGCTGCGCGTAACCTATG

a All primers were developed in the course of the present study.

### Neutrophil purification.

Human neutrophils were obtained from healthy adult donors using approved IRB protocol 16-012812 at the Children’s Hospital of Philadelphia. Whole blood was collected in Vacutainer K2 EDTA (K2E) Plus blood collection tubes (BD, Franklin Lakes, NJ). Neutrophils were isolated as previously described (43). Briefly, neutrophils were purified using 3% dextran sedimentation for 30 min at room temperature (RT) to separate red blood cells (RBCs) from the leukocyte-rich supernatant. The supernatant was transferred into a new conical tube, and 1 volume of Hypaque Ficoll (Sigma-Aldrich, St. Louis, MO) was added underneath the supernatant. The sample was centrifuged at 300 × *g* for 30 min to pellet the neutrophils. The remaining RBCs were subjected to hypotonic lysis with cold double-distilled water, and the isotonicity was restored by the addition of RPMI 1640 medium (Lonza, Walkersville, MD), without phenol red, supplemented with 10 mM HEPES (Sigma-Aldrich, St. Louis, MO). Neutrophils were pelleted and resuspended in RPMI 1640 medium-HEPES for experimental use.

### Neutrophil-killing assay.

Killing assays with neutrophils were performed as previously described (43). Briefly, neutrophils isolated from separate donors were diluted to 2.0 × 10^6^ cells/ml, and 500 μl was seeded onto 24-well plates in RPMI 1640 medium-HEPES with 1% human serum albumin (HSA) (Alfa Aesar, Ward Hill, MA) or 1% normal human serum (NHS) (Immucor, Norcross, GA). Bacteria were resuspended to an optical density at 600 nm (OD_600_) of ∼0.8 in phosphate-buffered saline (PBS) and added at both a high and low multiplicity of infection (MOI), 10:1 or 1:10, respectively, and centrifuged at 1,000 rpm for 2 min. Samples were incubated for various times at 37°C with 5% CO_2_. After incubation, the supernatant was removed and placed in 1.5-ml reaction tubes. Neutrophils were lysed using cold double-distilled water, wells were scraped, and the lysate was added to the corresponding collected supernatants. Serial dilutions of the inoculum and reaction samples were plated on chocolate agar plates and incubated overnight at 37°C with 5% CO_2_ to determine CFU counts (limit of detection for plating, 20 CFU). To perform inhibition assays, neutrophils were incubated with 10 μg/ml cytochalasin D (Life Technologies Corp., Frederick, MD), 10 mM *N*-acetylcysteine (Millipore/Sigma, Burlington, MA), 4 μM diphenyleneiodonium (Millipore/Sigma, Burlington, MA), or 1× protease inhibitor cocktail (Roche Diagnostics, Indianapolis, IN) for 30 min prior to infection with bacteria.

### Oxidative burst studies.

To detect intracellular and extracellular reactive oxidative species (ROS) generated by neutrophils, the neutrophils (isolated from separate donors) were diluted to 2.0 × 10^5^ cells/ml and seeded onto a white tissue culture-treated 96-well microtiter plate with 1% HSA or 1% NHS as previously described (44, 45). The bacteria were resuspended to an OD_600_ of ∼0.8 in phosphate-buffered saline (PBS) and added at an MOI of 10:1 to wells in the presence of 50 μM luminol. The microtiter plate was centrifuged at 100 × *g* for 2 min, and the relative amount of ROS generated by neutrophils was detected at 5-min intervals over 60 min by luminescence. Phorbol 12-myristate 13-acetate (PMA) (Alfa Aesar, Wardhill, MA) at a concentration of 40 ng/ml was used as a positive control for the generation of ROS by neutrophils.

### Immunofluorescence microscopy.

To visualize binding and phagocytosis of bacteria by neutrophils, neutrophils (isolated from separate donors) were diluted to 2.0 × 10^6^ cells/ml and seeded onto tissue culture-treated glass coverslips with 1% HSA or 1% NHS (45, 46). Bacteria were resuspended to an OD_600_ of ∼0.8 in PBS, stained with 5 μg/ml CFSE (Tonbo Biosciences, San Diego, CA) for 20 min at 37°C, and washed twice with PBS. CFSE-stained bacteria were added at an MOI of 10:1 and centrifuged at 1,000 rpm for 2 min. Samples were incubated for 30 min at 37°C with 5% CO_2_. Cells were washed twice with PBS to remove nonadherent bacteria and were fixed at RT for 15 min using 4% paraformaldehyde in PBS. Samples were incubated with anti-K. kingae antibody (1:500) overnight at 4°C. The anti-K. kingae antibody was generated against an acetone powder of K. kingae strain KK01 whole bacteria. Cells were washed twice with PBS and incubated with a 1:500 dilution of goat anti-guinea pig IgG DyLight 649-conjugated antibody (Rockland, Limerick, PA).

Visualization of bacteria-neutrophil association was performed by microscopy using a Nikon Eclipse Ni-E (Nikon Instruments, Inc., Melville, NY) with either a 20× objective or 60× oil objective. For quantification, 100 neutrophils per replicate were chosen at random for analysis; the cell-associated and extracellular bacteria were counted by a blinded reader.

### Antimicrobial peptide bactericidal assays.

K. kingae strains were grown on chocolate agar plates and then resuspended in PBS. Samples were diluted in PBS to obtain a final inoculum of approximately 4.0 × 10^3^ CFU/0.1 ml. The respective inocula were mixed with polymyxin B (Alfa Aesar, Ward Hill, MA), HNP-1 to HNP-3 (MilliporeSigma, Burlington, MA), or LL-37 (Bachem, Torrance, CA) at various concentrations and incubated at 37°C with 5% CO_2_ for 30 min. After incubation, 9 mM MgCl_2_ was added to the reaction mixture prior to plating. Serial dilutions of the inoculum and reaction samples were plated on chocolate agar plates and incubated overnight at 37°C with 5% CO_2_ to determine the CFU counts (limit of detection for plating, 20 CFU).

### Ethics statement.

Blood samples for neutrophil isolation were obtained from healthy adult donors that provided oral informed consent under approved Children’s Hospital of Philadelphia Institutional Review Board (CHOP IRB) protocol 16-012812 in compliance with all institutional and federal guidelines. Due to the nature of the study, the CHOP IRB indicated that oral consent was appropriate and approved its use. Oral informed consent from each study participant was recorded on a copy of the Verbal Consent Form, which was maintained in the study records.

### Statistical analysis.

Statistical analyses were performed with GraphPad Prism (version 7.0a) software for Mac (GraphPad Software, San Diego, CA), where a *P* value of <0.05 was considered statistically significant. The specific statistical tests used for each experiment are specified in the figure legends.
