# Supplementary figures and images for: Kingella kingae Surface Polysaccharides Promote Resistance to Neutrophil Phagocytosis and Killing
Source: mBio. 2019 Jun 25;10(3):e00631-19. doi: 10.1128/mBio.00631-19 (PMC6593399; doi:10.1128/mBio.00631-19)

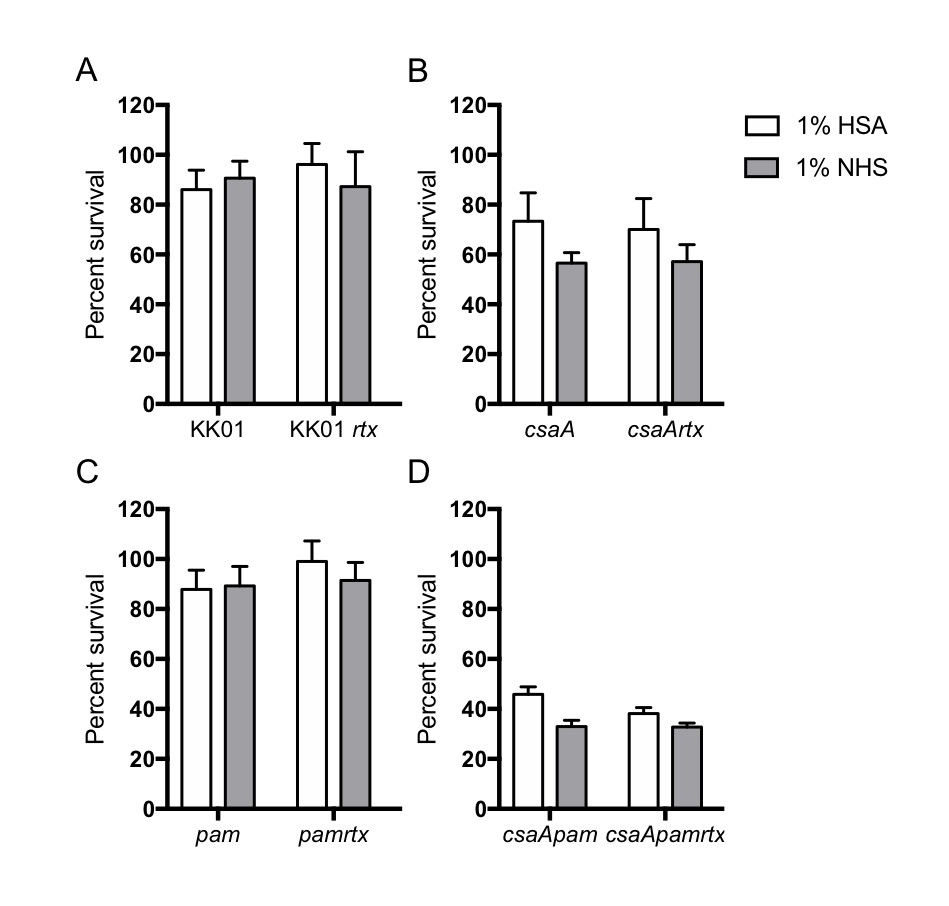

Supplement: FIG S2 [file mBio.00631-19-sf002.tif]
